# *Lactobacillus plantarum* Strain Ln4 Attenuates Diet-Induced Obesity, Insulin Resistance, and Changes in Hepatic mRNA Levels Associated with Glucose and Lipid Metabolism

**DOI:** 10.3390/nu10050643

**Published:** 2018-05-19

**Authors:** Eunjung Lee, So-Ra Jung, So-Young Lee, Na-Kyoung Lee, Hyun-Dong Paik, Seong-Il Lim

**Affiliations:** 1 Research group of Traditional Food, Korea Food Research Institute, Wanju 55365, Korea; jarasasa2@gmail.com; 2 Research Group of Natural Materials and Metabolism, Korea Food Research Institute, Wanju 55365, Korea; sylee09@kfri.re.kr; 3 Department of Food Science and Biotechnology of Animal Resources, Konkuk University, Seoul 05029, Korea; lnk11@konkuk.ac.kr (N.-K.L.); hdpaik@konkuk.ac.kr (H.-D.P.); 4 Research Group of Healthcare, Korea Food Research Institute, Wanju 55365, Korea

**Keywords:** lactobacillus plantarum Ln4, obesity, high fat diet, insulin resistance, metabolic disorder, insulin signaling

## Abstract

The prevalence of obesity and associated metabolic disorders, including diabetes and cardiovascular disease, is rapidly becoming a severe global health problem. Recent reports have suggested that the alteration of the gut ecosystem through the consumption of probiotics and fermented foods, such as yogurt and Kimchi, can significantly impact obesity and Type 2 diabetes (T2D)-related biomarkers. In this study, we screened over 400 strains of lactic acid bacteria (LAB) that were isolated from fermented foods to identify potent anti-obesogenic and diabetic probiotics in vitro. Of the strains tested, *Lactobacillus plantarum* Ln4 (Ln4), which was obtained from napa cabbage kimchi, significantly reduced lipid accumulation and stimulated glucose uptake in 3T3-L1 adipocytes. Oral administration of Ln4 reduced weight gain and epididymal fat mass in mice fed on a high-fat diet (HFD). Total plasma triglyceride level was significantly lower in mice that were treated Ln4 as compared with mice fed HFD. The protein levels of adipokines such as C-reactive protein (CRP), insulin-like growth factor binding proteins-3 (IGFBP-3), and monocyte chemoattractant protein-1 (MCP-1) decreased in white adipose tissues of Ln4-treated mice. Furthermore, these mice exhibited a significant reduction of insulin resistance index (HOMA-IR) and the improvement of glucose tolerance (OGTT) and insulin response (ITT) following Ln4 administration. This was associated with changes in several hepatic gene expressions (increased mRNA levels of IRS2, Akt2, AMPK, LPL, and reduced CD36) that regulate glucose and lipid metabolism. Taken together, these results indicate that in vitro and in vivo Ln4 treatment attenuates diet-induced obesity and T2D biomarkers, highlighting the potential of Ln4 as a therapeutic probiotic agent for metabolic disorders.

## 1. Introduction

The prevalence of obesity has risen steeply over the past two decades, and it has become a significant global health issue [1]. The proper management of obesity is critical to prevent the development of obesity-associated metabolic disorders, including insulin resistance, diabetes, and cardiovascular disease, which increase patient mortality [2,3]. Lipid accumulation and increased systemic inflammation in obesity is known to trigger an imbalance of energy homeostasis and abnormal cellular responses to insulin, leading to insulin resistance and type 2 diabetes (T2D) [4,5]. Although a number of therapies have been developed to treat them, the disease remains largely irreversible, with many patients failing to respond satisfactorily to treatment, underlining the importance of prevention at the early stages of obesity and insulin resistance [6].

The gut microbiome is composed of a thousand of bacterial species that are encoding approximately 3.3 million genes and largely shared 160 species individually [7]. It is also known to have a profound impact on host physiology and pathology. The diversity and stability of the gut microbiome in host organisms can, in turn, be affected by diverse environmental factors, including food consumption, which ultimately influences host metabolism [8,9,10]. Changes to the gut microbiota are associated with the development of obesity and T2D. Furthermore, targeting the gut microbiome using probiotics and prebiotics has been suggested as a new therapeutic approach against obesity and metabolic disorders [11].

To ameliorate obesity and associated metabolic syndromes, several therapeutic agents using microorganisms have been investigated. Previous studies have shown an association between the consumption of fermented foods containing an abundancy of live probiotic microbes, and reduced obesity and improvement in metabolic parameters [12,13,14]. After fermentation using the specific probiotics, several staple foods, including milk, soybean, and vegetables have reported exhibited properties, as consistent with the lowering of obesity and diabetic effects [15,16,17,18,19]. Among them, Kimchi is a traditional fermented food in Korea that uses napa cabbage, white radish, red pepper, and green onion. It has been demonstrated that its consumption reduces weight gain and improves the metabolic parameters in obese patients [13,20,21]. According to results from whole-genome sequences of the metagenome, abundant microbial populations are in kimchi with dominant members of genera, especially *Leuconostoc*, *Weissella*, and *Lactobacillus* [22]. The complexity of kimchi microflora arises from the unsterilized vegetables from which it is made, and these microorganisms affect both the flavor and the chemical changes throughout the fermentation period. Recent studies have drawn associations between improvements in metabolic syndrome and the consumption of fermented kimchi and lactic acid bacteria (LAB)-associated changes in gut microbiota, which induce lipid-lowering effects [13,23,24]. Beyond the role for kimchi as a fermented food for the treatment of obesity, artificially-fermented kimchi using specific LAB strains or LAB alone have been suggested as therapeutic products for the treatment of obesity [25,26]. Although several previous studies have shown that LAB from kimchi or kimchi fermented with specific LAB ameliorates obesity [24,27,28], these findings have been limited in scope as only specific strains or materials were used. Because potential probiotics in fermented foods are so abundant, the efficient in vitro screening is needed to identify probiotics having anti-obesity effects. The adipocyte differentiation and adipogenesis in 3T3-L1 cells is the most commonly used in vitro model to examine the effect on obesity [29]. However, the treatment of live probiotics in 3T3-L1 cells is limited due to contamination, so that several assumptions are needed for the experiment. The tolerance for gastric and bile acid, adhesiveness to intestinal epithelial cells, non-cytotoxicity, and the susceptibility to antibiotics are basically required for probiotics, which means the higher possibility to survive in the gastrointestinal (GI) tract [30,31]. When probiotics are orally administrated, it may be difficult for live probiotics to directly reach the adipose tissue. In that case, the components of the dead cells or the metabolites may have an effect on adipocytes. If the probiotics are colonized in the GI tract, they could produce a variety of metabolites and effects on peripheral tissues. Although in vitro studies of obesity and related diseases may provide an efficient selection and specific mechanistic action, their applicability to in vivo test is relatively limited by the existing differences in physiological metabolism. Thus, in vivo experiments using candidate probiotics that passed the in vitro test should be evaluated. In the current study, we examined a total 426 strains of LABs isolated from fermented food to discover the anti-obesity and diabetic probiotics through the in vitro test, and selected the LAB, *Lactobacillus plantarum* strain Ln4 having inhibitory effect on adipocyte differentiation and the stimulatory ability on glucose uptake in 3T3-L1 cells. The inhibitory effect of Ln4 administration on obesity and insulin resistance was confirmed in high-fat diet fed mice, highlighting the potential of Ln4 that was isolated from Kimchi as a potential therapeutic probiotic agent for obesity and related metabolic disorders.

## 2. Materials & Method

Strains. For this study, 426 strains preliminarily screened were collected from mainly Korean Culture Center of Microorganisms (KCCM), Dankook University, Gachon University, and Korea Food research Institute (KFRI). Of the strains screened, the selected 22 strains were kindly provided from Prof. Hyun-dong Paik; *Lactobacillus plantarum* (*n* = 9), *Lactobacillus brevis* (*n* = 7), *Leuconostoc lactis* (*n* = 2), *Lactobacillus fermentum* (*n* = 1), *Lactobacillus buchneri* (*n* = 1), and *Leuconostoc citreum* (*n* = 1), *Pediococcus pentosaceus* (*n* = 1). A strain Ln4 that was isolated from napa cabbage (KCCM11897P) was identified as *Lactobacillus plantarum* by 16S rRNA sequencing and grown in lactobacilli MRS (MRS) broth [31]. For in vitro studies, Ln4 cells were produced by sterilization by autoclave and called as heat-killed cell. Cultured MRS broth was freeze-dried and referred to freeze-dried broth.

Cell culture and differentiation. 3T3-L1 murine preadipocytes were obtained from the American Type Culture Collection and grown in Dulbecco’s modified Eagle’s medium (DMEM; GIBCO, Carlsbad, CA, USA) with 10% fetal bovine serum (FBS; GIBCO, Carlsbad, CA, USA), 100 unit/mL penicillin (Sigma-Aldrich, St. Louis, MO, USA), and 100 µg/mL streptomycin (Sigma-Aldrich, St. Louis, MO, USA) at 37 °C in an atmosphere of 5% CO_2_. Adipocyte differentiation was undertaken in culture with MDI mixture containing 5 μM dexamethasone (Sigma-Aldrich, St. Louis, MO, USA), 0.5 mM 3-isobutyl-1-methylxanthine (Sigma-Aldrich, St. Louis, MO, USA), and 167 nM insulin (Sigma-Aldrich, St. Louis, MO, USA) in 10% FBS/DMEM for 2 days. The MDI medium was changed to 10% FBS/DMEM containing 167 nM insulin for two days and was replaced with 10% FBS/DMEM for the formation of lipid droplets within adipocytes. Fully differentiated cells were subjected to Oil Red O staining to quantify the intracellular lipid contents by measuring the absorbance at 519 nm using a microplate reader (Varioskan, Thermo Electron Co., Waltham, MA, USA) after dissolving Oil Red O solution (Sigma-Aldrich, St. Louis, MO, USA) in isopropyl alcohol (Sigma-Aldrich, St. Louis, MO, USA) [32]. To compare the effect of 22 strains on lipid accumulation, 3T3-L1 pre-adipocytes were seeded in 96-well plate. Microphotographs were obtained from differentiated adipocytes seeded in a six-well plate by optical microscopy (×200).

Glucose uptake in 3T3-L1 adipocytes. Fully differentiated 3T3-L1 adipocytes were washed with phosphate-buffered saline (PBS; GIBCO, Carlsbad, CA, USA) twice and incubated in low glucose DMEM (1000 mg/L of glucose; GIBCO, Carlsbad, CA, USA) for 1 h in a CO_2_ incubator. Then, cells were treated with heat-killed cells or freeze-dried broth from 3 strains or 100 nM of insulin (as a positive control) for 1 h. To quantify glucose uptake into the cells, a fluorescent glucose analog, 2-(*N*-(7-nitrobenz-2-oxa-1,3-diazol-4-yl) amino)-2-deoxyglucose (2-NBDG; Invitrogen, Carlsbad, CA, USA) was used [22]. After co-incubating with 20 µM of 2-NBDG for 4 h, the cells were washed three times in PBS and lysed with 70 µL of 0.1M potassium phosphate buffer containing 1% Triton X-100 in a 96-well plate. 30 µL of dimethyl sulfoxide (DMSO; Sigma-Aldrich, St. Louis, MO, USA) was added to each well, and intracellular glucose was quantified by detecting fluorescence (λ_ex_ = 466 nm, λ_em_ = 587 nm).

Animals. Male C57BL/6J mice were purchased from Japan SLC Inc. (Shizuoka, Japan) and housed under controlled temperature (23 °C) and lighting (12 h of light, 7 a.m. to 7 p.m.; 12 h of dark, 7 p.m. to 7 a.m.) conditions. The animal studies were approved by the Institutional Animal Care and Use Committee of Korea Food Research Institute (approval date: 01-11-2017, approval number: KFRI-M-17004). At the age of 12 weeks, the mice were randomly selected and divided into three groups (5–7 mice per group) and fed normal chow diet (Harlan Teklad 2018 diet; Harlan Laboratories, Indianapolis, IN, USA) or high-fat diet (HFD, Harlan Teklad TD 07011; 55% kcal from fat; Harlan Laboratories, Indianapolis, IN, USA) *at libitum* for five weeks. For Ln4 group, 5 × 10^8^ CFU (colony forming unit) of live Ln4 was daily administrated by oral gavage in 0.2 mL of distilled water (DW) and concurrently fed HFD. Control and HFD groups also consumed 0.2 mL of vehicle (DW) by oral gavage. After four weeks of treatment, plasma was obtained from tail vein of mice fasted overnight to measure plasma lipid and glucose tolerance (OGTT). Three days afterward, mice were fasted again for 5 h to perform insulin tolerance test (ITT) and to measure plasma glucose and insulin. During the experiment, body weight and food intake were measured weekly. After the end of the experiment at five weeks, mice were sacrificed and taken tissues (epididymal fat, brown adipose tissue, liver, pancreas, and heart) under anesthesia. Tissues were frozen at −70 °C or fixed in 4% paraformaldehyde solution.

Plasma analysis. After four weeks of HFD feeding, mice were fasted overnight and were bled by their tail vein into a heparinized tube. Plasma was obtained after centrifugation at 10,000× *g* for 10 min from blood and used for the measurement of total triglycerides (TG), high density lipoproteins (HDL)-cholesterol, and low density lipoproteins (LDL)-cholesterol. To determine plasma glucose and insulin levels, mice were fasted for 5 h, and then bled by their tail vein into a heparinized tube. Plasma glucose was measured by the glucometer (Accu-Chek Performa, Roche Diagnostics, Indianapolis, IN, USA). Plasma insulin levels were determined by ELISA (ALPCO, Salem, NH, USA), and the total TG was measured using a serum triglyceride determination kit (Sigma-Aldrich, St. Louis, MO, USA). HDL- and LDL-cholesterol (Biovision Inc., Milpitas, CA, USA) were analyzed in accordance with the manufacturer’s instructions.

Measurement of insulin resistance in mice. To analyze insulin resistance in live mice, HOMA-IR calculation, oral glucose tolerance test (OGTT), and an insulin tolerance test (ITT) were carried out in mice after four weeks of treatment. HOMA-IR was calculated using the following equation [33]; HOMA-IR = fasting glucose (mg/dL) × fasting insulin (μU/mL)/405. To carry out the OGTT assay, mice were fasted overnight and were orally administered by 1 g/kg of glucose. The plasma glucose levels were measured at 0, 15, 30, 60, 90, and 120 mins from the tail vein by glucometer. For ITT experiments, mice were fasted for 5 h in the early morning and were administered with 0.75 IU/kg of insulin by intraperitoneal injection. Plasma glucose levels were also measured at 0, 15, 30, 60, and 90 mins from the tail vein.

H&E staining. Liver tissues from mice were fixed in 4% paraformaldehyde solution overnight and were embedded in paraffin. The sections (5 μm thick) on silane-coated slides were deparaffinized twice with xylene and dehydrated through a graded alcohol chamber. After hematoxylin and eosin (H&E) staining, slides were washed and mounted with coverslip. The representative pictures were taken from 5–7 individual liver samples in each group (×200 magnification) using microscopy (Olympus BX-43, Olympus Optical, Tokyo, Japan).

Protein array analysis. 300 mg of epidydimal fat tissues from mice were used in a mouse adipokine array (R&D Systems, Minneapolis, MN, USA), according to the manufacturer’s instructions. Analysis and quantification of the array membrane was completed using Alliance Mini HD9 (UVITEC, Cambridge, UK) and Image J software (1.49v, NIH, Bethesda, MD, USA), respectively. All of the quantified data were normalized by intensity of reference spots in each membrane, as followed by manufacturer’s instruction.

Quantitative real-time PCR. Liver tissue samples from mice were extracted using the TRIzol method (Invitrogen, Carlsbad, CA, USA), and synthesized cDNA from 2 μg of total RNA via iScript^TM^ cDNA synthesis kit (Biorad, Hercules, CA, USA). As shown in Table 1, the mRNA levels that were related to glucose and lipid metabolism were determined using specific primers and real-time PCR was performed using a Fast SYBR green Master Mix kit (Life Technologies, Gaithersburg, MD, USA) on a StepOne plus instrument (Applied Biosystems, Foster City, CA, USA). For normalization and heatmap analysis of raw data, ExpressionSuite software (v.1.0.3, Applied Biosystems, Foster City, CA, USA) was used.

Statistical analysis. All data are expressed as mean ± SEM. The data from adipocyte differentiation and glucose uptake assay were analyzed by one-way ANOVA, followed by Dunnett’s test (*, *p* < 0.05; **, *p* < 0.01; ***, *p* < 0.001 vs. MDI). In vivo experiments were analyzed by one-way or two-way repeated measures ANOVA, followed by Tukey’s test. For statistical significance, a probability value of *p* < 0.05 was used and marked as * (*, *p* < 0.05; **, *p* < 0.01; ***, *p* < 0.001 vs. Control) or # (#, *p* < 0.05; ##, *p* < 0.01; ###, *p* < 0.001 vs. HFD). All of the analyses were performed using Statistical Analysis Software (version 9.2, SAS Inc., Cary, NC, USA).

## 3. Results

**Figure 1:**
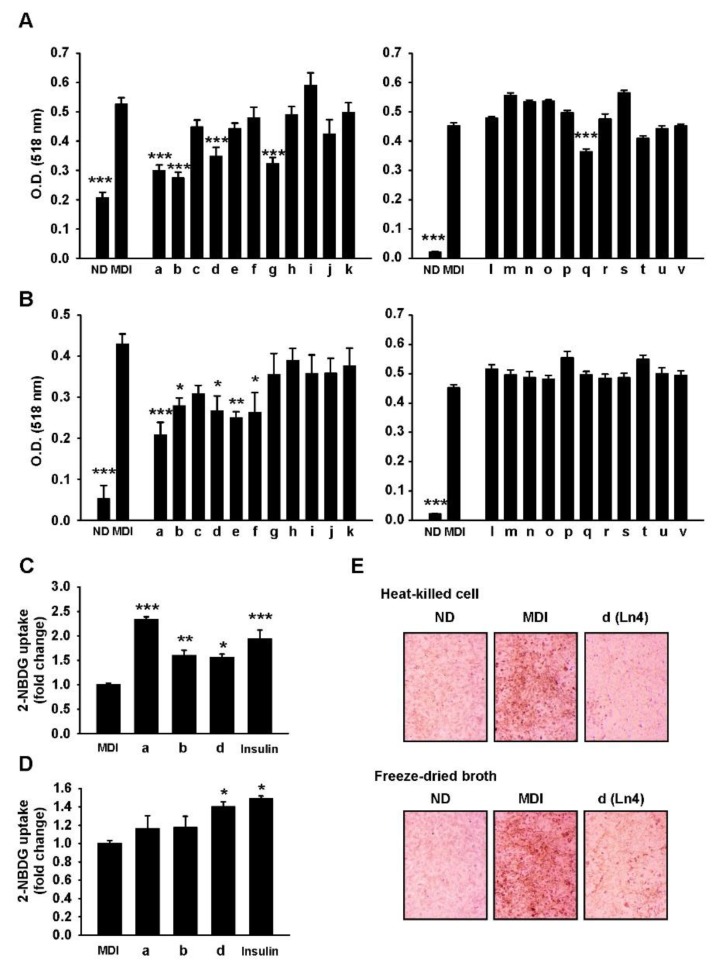
Ln4 treatment inhibits lipid accumulation and enhances glucose uptake in 3T3-L1 adipocytes. (**A**,**B**) 3T3-L1 pre-adipocytes were seeded in 96-well plates and incubated with heat-killed cells (**A**) or freeze-dried broth (**B**) of 22 strains of lactic acid bacteria (LAB) (100 μg/mL, designated by a-v) during differentiation to adipocytes. Lipid droplets were detected by Oil Red O staining and measured by absorbance at 518 nm after dissolving in 2-propanol. *, *p* < 0.05; **, *p* < 0.01; ***, *p* < 0.001 vs. nM insulin (MDI) (ANOVA followed by Dunnett’s test) (**C**,**D**) Fully differentiated 3T3-L1 adipocytes were incubated with heat-killed cells (**C**), freeze-dried broth (**D**) of three strains of LAB (100 μg/mL) or insulin as a positive control (100 nM). Glucose uptake were detected by fluorescence-labeled glucose analog (2-NBDG) after 4 h incubation as described in Materials & Method section. Each bar represents mean ± SEM followed by 3 independent experiments. Asterisks (*, **, and ***) indicate statistical significance (one-way ANOVA/Dunnett’s test, *p* < 0.05, 0.01, and 0.001, respectively vs. MDI). (**E**) 3T3-L1 pre-adipocytes were seeded in 6-well plates and incubated with heat-killed cells (*upper*) and freeze-dried broth (*bottom*) of Ln4. The representative pictures of stained lipid droplets (×200) are presented. ND, non-differentiated group; MDI, differentiated group; O.D., Optical density; a, *Lactobacillus brevis* B151; b, *Lactobacillus fermentum* KCCM200060; c, *Lactobacillus plantarum* G72; d, *Lactobacillus plantarum* Ln4; e, *Lactobacillus plantarum* Lb41; f, *Lactobacillus brevis* G1; g, *Lactobacillus brevis* KCCM200080; h, *Lactobacillus brevis* KCCM200054; i, *Leuconostoc citreum* S.Pum19; j, *Pediococcus pentosaceus* SC28; k, *Lactobacillus brevis* KCCM200019; l, *Leuconostoc Lactic* KCCM202361; m, *Leuconostoc lactis* KCCM202369; n, *Lactobacillus plantarum* KCCM200656; o, *Lactobacillus plantarum* KCCM200693; p, *Lactobacillus plantarum* KCCM200650; q, *Lactobacillus plantarum* KCCM202303; r, *Lactobacillus plantarum* KCCM200655; s, *Lactobacillus plantarum* KCCM200661; t, *Lactobacillus brevis* KCCM202302; u, *Lactobacillus brevis* KCCM202399; v, *Lactobacillus Buchneri* KCCM200793.

L. plantarum Ln4 inhibits adipogenesis and stimulates glucose uptake in 3T3-L1 adipocytes. To identify potential probiotic lactic acid bacteria (LAB) that were isolated from traditional fermented food, in vitro screening assays were performed using 462 strains that were tested for tolerance to artificial gastric acid and bile salts. Of the strain tested, 69 strains exhibited resistance to artificial gastric acid (0.3% pepsin, pH 2.5) and bile salts (0.3% Oxgall), defined as having a survival rate of over 80%. These were sequentially tested for the non-productivity of β-glucuronidase, adhesiveness to intestinal epithelial cells in HT-29 human colon adenocarcinoma (>1%), cell cytotoxicity in MRC-5 human fibroblast cells (>20%), and susceptibility to ampicillin, tetracycline, chloramphenicol, and doxycycline. Only 22 strains, including *Lactobacillus plantarum, Lactobacillus brevis, Lactobacillus fermentum, Leuconostoc lactis, Lactobacillus buchneri, Leuconostoc citreum,* and *Pediococcus pentosaceus* were qualified (Figure 1). To determine the effect of these 22 strains on lipid accumulation in vitro, 3T3-L1 pre-adipocytes were treated with heat-killed cells (Figure 1A) and freeze-dried broth (Figure 1B). After differentiation, lipid contents was measured by Oil Red O (ORO) staining, and was found to be significantly increased in differentiated cells (labeled MDI) when compared to non-differentiated (ND) cells. The three strains of LAB, including *L. brevis* B151 (a), *L. fermentum* KCCM200060 (b), and *L. plantarum* Ln4 (d) exhibited reduced lipid accumulation by both heat-killed cells and freeze-dried broth. Glucose uptake assays in 3T3-L1 mature adipocytes showed that there was significant stimulation of intracellular glucose absorption in adipocytes in the presence of heat-killed cells (Figure 1C) and freeze-dried broth (Figure 1D) of *L. plantarum* Ln4 (Ln4). Through these results, Ln4 was eventually selected as a candidate probiotic LAB with anti-obesity and diabetic potential. 

**Figure 2:**
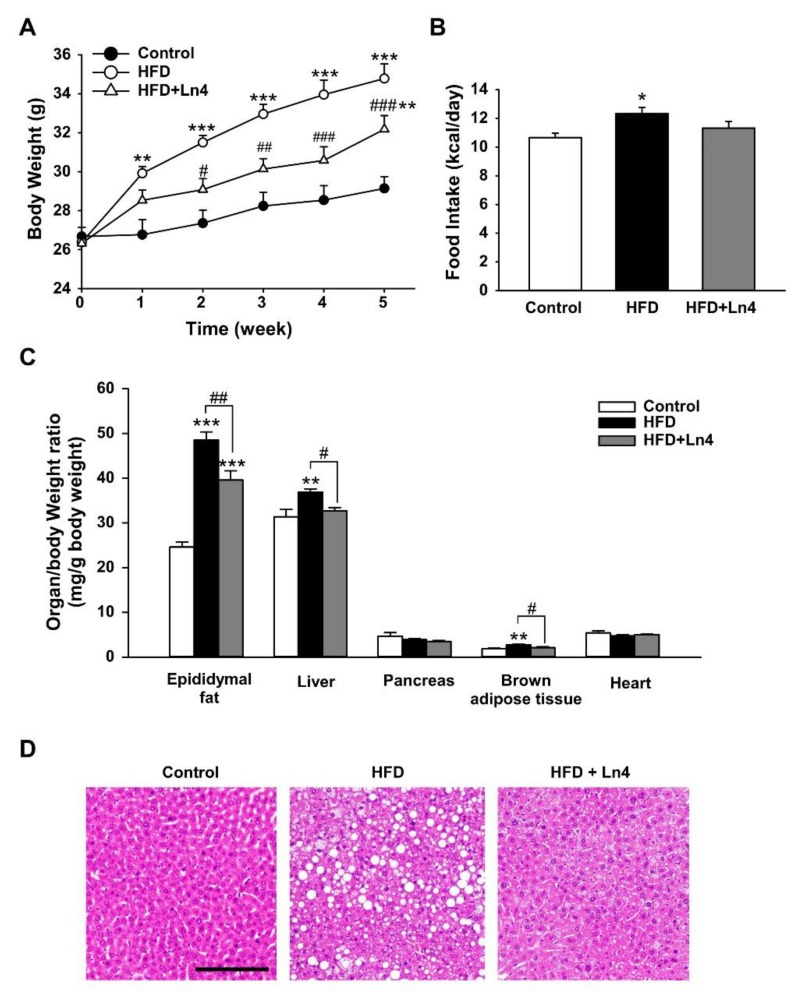
Oral administration of Ln4 reduces high-fat diet-induced weight gain and organ weights in mice. (**A**) Body weight was measured weekly and presented as mean ± SEM (*n* = 5–7). The statistical significance was analyzed by Tukey’s test after two-way repeated measures ANOVA (**, *p* < 0.01; ***, *p* < 0.001 vs. Control and #, *p* < 0.05; ##, *p* < 0.01; ###, *p* < 0.001 vs. HFD). (**B**) The averaged food intake of each group was calculated and shown as mean ± SEM. The asterisks mean the statistical significance with Control group (*, *p* < 0.05). (**C**) The tissues were weighed and normalized by the body weight of each mouse. Each graph show mean ± SEM. A probability value was marked as asterisks (**, *p* < 0.01; ***, *p* < 0.001 vs. Control) or # (#, *p* < 0.05; ##, *p* < 0.01 vs. HFD) analyzed using Tukey’s test after one-way ANOVA. (**D**) Histochemical staining of liver from mice. The morphological changes and the lipid accumulations in the liver were performed by H&E staining. Representative pictures were taken from 5–7 independent tissue samples in each group (×200 magnification). Scale bar, 100 μm.

Administration of Ln4 inhibits diet-induced weight gain and lipid accumulation in mice. To investigate the effect of Ln4 on diet-induced obesity in vivo, C57BL/6J mice were randomly divided into three groups. They were fed on a chow diet (control group) or HFD with daily oral treatment of 5 × 10^8^ live Ln4 cells (Ln4 group) or vehicle (HFD group). After five weeks of feeding and treatment, the average body weight of the mice in the HFD group was significantly increased when compared to the controls (Figure 2A). By oral gavage of live Ln4, increased body weight by HFD feeding was reduced without changes in energy intake (Figure 2B). The increased organ weights, including epidydimal fat, liver, and brown adipose tissue by HFD feeding also resulted in significant reductions following Ln4 administration (Figure 2C), while the averaged weights of the heart and pancreas were not influenced by Ln4 treatment. The levels of plasma insulin and total triglyceride (TG) were significantly decreased in the Ln4 group when compared to the HFD group (Table 2). 5-h fasted plasma glucose levels showed a slight decreasing tendency in the Ln4 group, but statistical significance was weak (*p* = 0.063). Through histological examinations of H&E stained liver sections, we confirmed that mice fed HFD for five weeks developed a fatty liver phenotype showing morphological changes that were caused by lipid deposition (Figure 2D). Ln4 administration also alleviated fatty liver, indicating the inhibitory effect of Ln4 on hepatic lipid accumulation in mice fed HFD. These results show that Ln4 attenuates HFD-induced weight gain and hyperlipidemia in a mouse model.

**Figure 3:**
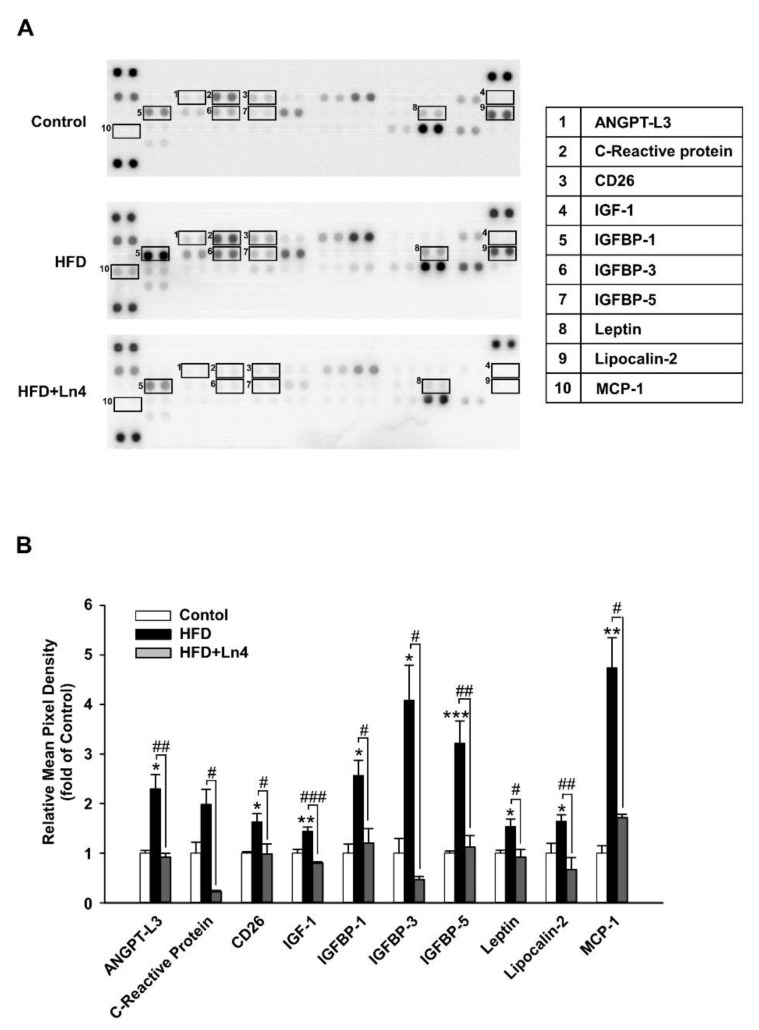
Adipokine array analysis of white adipose tissue from mice. (**A**) Epidydimal fat tissues from mice (*n* = 4) were prepared as described in Materials & Method section. Representative proteins identified are marked as square, and each number presents different proteins described in the table on the right side. (**B**) Protein array membranes were quantified using Image J software. Selected proteins showing statistical group difference between HFD and HFD+Ln4 (#, *p* < 0.05; ##, *p* < 0.01; ###, *p* < 0.001, ANOVA, followed by Tukey’s test) are presented as mean ± SEM. * *p* < 0.05, ** *p* < 0.01, *** *p* < 0.001, significant differences compared with Control. ANGPTL3, angiopoietin-like 3; CD26, dipeptidyl peptidase-4; IGF, insulin-like growth factor; IGFBP, IGF-binding protein; MCP-1, monocyte chemoattractant protein 1.

*Ln4 treatment causes changes in adipokine profile of adipose tissue*. To determine the mechanistic effect of Ln4 on obesity, we performed a protein array analysis that was focused on adipokine levels using white adipose tissue samples (epidydimal fat) from mice in the HFD and Ln4 groups (Figure 3). The results showed the suppression of several adipokine proteins, including ANGPT-L3, C-reactive protein (CRP), leptin, lipocalin-2, monocyte chemoattractant protein-1 (MCP-1), and insulin-like growth factor binding proteins (IGFBPs), as a result of Ln4 treatment. Especially, HFD feeding caused a ~4-fold increase in the protein levels of IGFBP-3 and MCP-1 that was associated with glucose utilization and insulin resistance in adipose tissues. It almost restored to normal protein levels that were similar to control group by Ln4 administration. Thus, Ln4 administration alleviates HFD-induced abnormal adipokine parameters, and may potentially improve insulin sensitivity.

**Figure 4:**
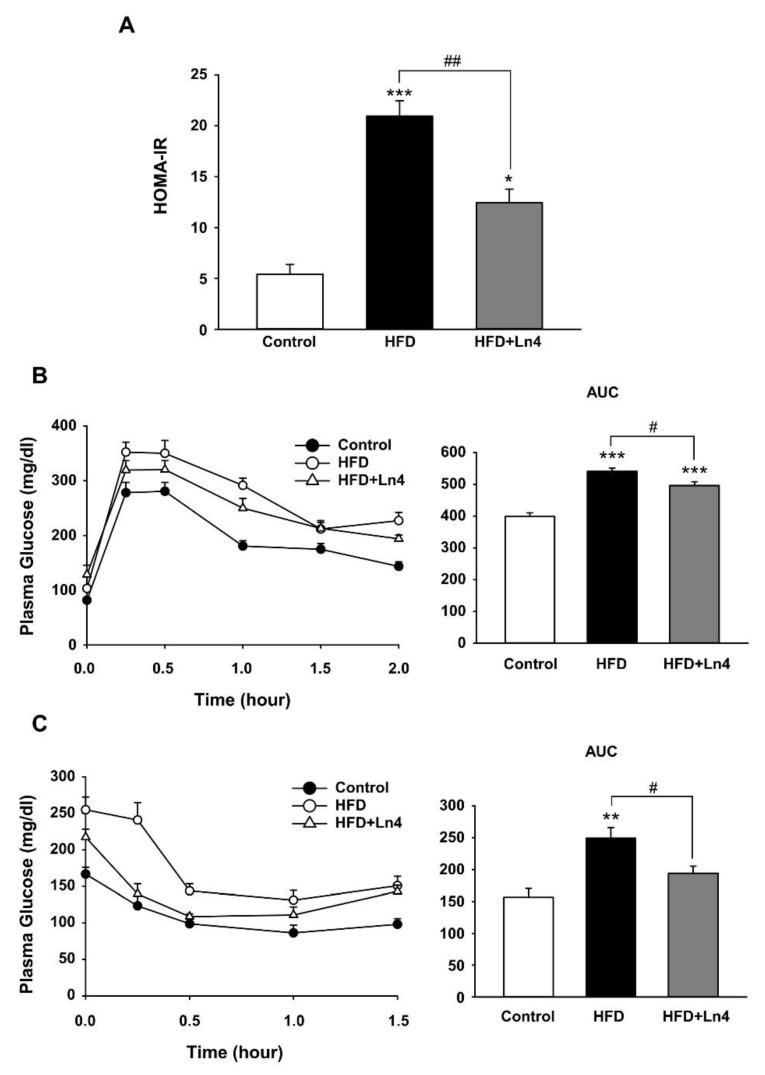
Effects of Ln4 supplementation on the homeostatic model assessment (HOMA) of insulin resistance (HOMA-IR), glucose tolerance (GTT), and insulin tolerance (ITT) in mice. (**A**) Averaged HOMA-IR index in each group. (**B**) Oral glucose tolerance test was performed in mice fasted overnight. Area under curve (AUC, *right*) is presented by bar graph (mean ± SEM) (**C**) Insulin tolerance test was conducted in 5-h fasted mice by measuring plasma glucose levels during i.p. insulin. AUC (*right*) is presented as mean ± SEM. Statistical significance was analyzed using one-way ANOVA followed by Tukey’s test and is shown with asterisks (*, *p* < 0.05; **, *p* < 0.01; ***, *p* < 0.001 vs. Control) or # (#, *p* < 0.05; ##, *p* < 0.01 vs. HFD).

Ln4 administration attenuates HFD-induced insulin resistance in mice. To verify the improvement in insulin sensitivity by Ln4, we measured insulin resistance index and conducted oral glucose tolerance tests (OGTT) and insulin tolerance tests (ITT) in mice. Homeostatic model assessment for insulin resistance index (HOMA-IR) is calculated by fasting glucose and insulin levels, and it was highly elevated in response to HFD feeding, up to 3.9-fold when compared to the control group (Figure 4A). Ln4 administration induced a 40.6% reduction to the increased HOMA-IR index resulting from HFD feeding. In Figure 4B, a rapid rise within 0.5 h and a gradual decline over 2 h for plasma glucose levels following the oral injection of glucose solution in mice was generally lowered by Ln4 administration as compared to the HFD group. Averaged area under curve (AUC) values for the OGTT data also showed upregulation by HFD feeding when compared to the control group, and it was significantly reduced by an oral gavage of Ln4. Moreover, reduced plasma glucose levels by intraperitoneal injection of insulin was blunted by HFD feeding, which was also recovered by Ln4 treatment (Figure 4C). AUC data from ITT also indicated statistical significance between the HFD- and Ln4-treated groups. Taken together, these results suggest that Ln4 administration improves glucose and insulin tolerance, and it attenuates HFD-induced insulin resistance in mice.

**Figure 5:**
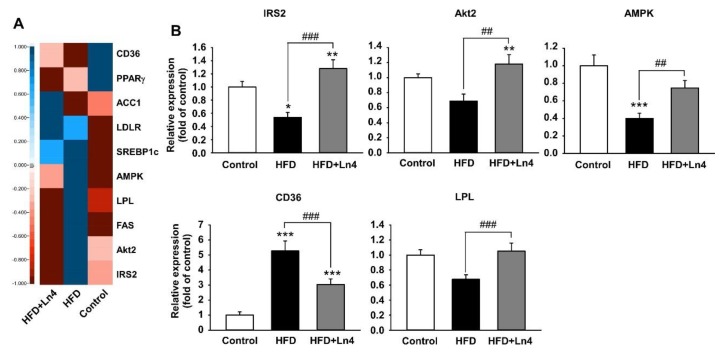
Effect of Ln4 administration on mRNA levels of hepatic genes associated with glucose and lipid metabolism. (**A**) Heatmap analysis for qPCR data from liver lysate. Five biological replicates were performed for each group. (**B**) Statistically significant changes on hepatic mRNA levels related glucose and lipid metabolism are presented by mean ± SEM is presented as mean ± SEM. Quantitative PCR data was tested for statistical difference using one-way ANOVA, followed by Tukey’s test (*, *p* < 0.05; **, *p* < 0.01; ***, *p* < 0.001 vs. Control or ##, *p* < 0.01; ###, *p* < 0.001 vs. HFD). PPARγ, peroxisome proliferator-activated receptor gamma; AMPK, AMP-activated protein kinase; CD36, cluster of differentiation 36 or platelet glycoprotein 4; IRS2, insulin receptor substrate 2; LPL, lipoprotein lipase; LDLR, low-density lipoprotein receptor; ACC1, acetyl-CoA carboxylase; FAS, fatty acid synthase; SREBP1c, sterol regulatory element-binding protein 1c; Akt2, protein kinase Bβ.

The exposure of Ln4 influences in the alteration of mRNA levels related glucose and lipid metabolism in liver. A decrease in the protein levels that were involved in nutrient metabolism and insulin signaling pathway and by HFD feeding in insulin-responsive organs including the liver, adipose tissue, and skeletal muscle can lead to the development of insulin resistance. The restoration of the function of these proteins is strongly associated with improvements in insulin sensitivity. Especially, glucose and lipid metabolism in the liver plays a crucial role in the pathogenesis of obesity, hepatic steatosis, and whole-body insulin resistance [34]. As shown in Table 2, the specific primers for quantitative real-time PCR were used to determine the mRNA levels that were involved in hepatic glucose and lipid metabolism (Figure 5). In the liver, metabolic factors that are associated with glucose and lipid metabolism, including IRS2, Akt2, and AMPK were significantly elevated by Ln4 treatment, while in HFD-fed mice, the hepatic mRNA levels of IRS2 and AMPK were reduced. CD36 plays a critical role in the development of fatty liver and showed greater mRNA level in the livers of HFD-fed mice than in the control group. The reduced mRNA level of CD36 in the liver was detected in the Ln4 group, consistent with improved fatty liver by Ln4 administration in mice fed HFD (Figure 2D). Also, the mRNA levels of lipoprotein lipase (LPL), which catalyzes the hydrolysis of plasma TG, was significantly increased in the livers of the Ln4 group. Taken together, our results suggest that Ln4 administration attenuates HFD-induced weight gain, hyperlipidemia, hepatic lipid accumulation, and insulin resistance by the regulating mRNA levels that are associated with glucose and lipid metabolism in the liver. It further suggests the possibility that Ln4 could be developed as a potential therapeutic agent for the treatment of obesity and diabetes.

## 4. Discussion

In recent years, probiotics have become an emerging platform for therapeutic and preventive agents against metabolic syndrome. Findings from experimental and clinical studies have supported the utility of probiotics against obesity and diabetes [35,36]. Despite the potential of probiotics and fermented foods that are enriched with them, there are limitations in its discovery and the development of preventive and therapeutic agents for obesity and metabolic syndrome. The microorganisms exhibit species-, and even strain-specific effects and different mechanistic actions, and this complexity requires efficient validation processes. In this study, we excluded 95% of total candidate probiotics isolated from fermented foods by primary in vitro screening and finally selected Ln4 by adipogenesis and glucose uptake in 3T3-L1 to development of probiotics having anti-obesity and diabetic effect. Of course, the failed LABs in this screening may show an anti-obesity effect in vivo. This is because in vitro experiments only test a very small part of the systemic mechanisms that control obesity. We conducted the additional in vitro experiments (assays for glycerol release and α-glucosidase inhibition), which are widely used for obesity, but we did not obtain meaningful results because of the relatively low discrimination between probiotics (data not shown). Instead, we found that both heat-killed and freeze-dried broth of Ln4 significantly reduced lipid accumulation and stimulated glucose uptake in 3T3-L1 cells. Live Ln4 administration orally in HFD-fed mice strongly attenuated weight gain and insulin resistance. Recent studies have suggested that even dead probiotic cells or their cell components containing metabolites can be beneficial for host health [37,38,39]. In addition, heat-killed LAB or freeze-dried broth has previously exhibited an inhibitory effect on adipogenesis in 3T3-L1 cells [40,41]. These results support the availability of using 3T3-L1 as an in vitro tool for the selection of probiotics with an anti-obesity and anti-diabetic effect.

In mice, Ln4 administration altered the mRNA levels that are associated with glucose and lipid metabolism and insulin-responsive signaling pathway. The data from adipokine array analysis in Figure 3 showed the increased the level of several adipokines such as IGFBPs, leptin, lipocalin-2, and MCP1 which are known to be involved in the pathogenesis of obesity and insulin resistance by HFD feeding. IGFBP-3 is highly expressed by hyperinsulinemia in obese subjects, and its infusion reduces glucose utilization and glycogen synthesis, which suggests an association between insulin resistance and high levels of IGFBP-3 [42,43]. Leptin and lipocalin-2, which are mainly produced in adipose tissues, are up-regulated in obesity and lead to the development of insulin resistance [44,45]. In addition, the adipocyte-specific overexpression of MCP-1 in mice induces the increased macrophage infiltration into adipose tissue, hepatic steatosis, and insulin resistance [46]. In this study, the increased protein levels of IGFBP-3, leptin, lipocalin-2, and MCP-1 by HFD feeding were significantly reduced in the adipose tissues of Ln4-administrated mice. These data indicate that Ln4 attenuates diet-induced obesity and the abnormal protein levels in adipose tissues that are associated with insulin action, as well as glucose and lipid metabolism.

In the liver, the alteration in glucose and lipid metabolism is linked to the pathogenesis of obesity, hepatic steatosis, and whole-body insulin resistance. IRS2/PI3K/Akt2 signaling plays an important role in insulin action to regulate glucose transport and lipid/glycogen synthesis, and AMPK is amaster regulator of lipid and protein synthesis and glucose transport [47]. These pathways are blunted by HFD feeding, which suggests that restoring the pathways is a critical strategy for insulin sensitivity. We confirmed that Ln4 administration induced the up-regulation of hepatic mRNA levels, including IRS2, Akt2, and AMPK, and these results correspond to the data of improving systemic insulin resistance in Ln4-treated mice. CD36 is directly associated with the development of fatty liver and insulin resistance by modulating lipid uptake in hepatocytes. Our data showed the 5.3-fold higher mRNA level of CD36 in the HFD group than in the Control group, and a ~50% of reduction by Ln4 administration. In addition, we found reduced liver weight, morphological recovery from fatty liver, and improved insulin sensitivity in Ln4-administrated mice, which correspond to the result from mRNA level of CD36.

Taken together, we have shown that Ln4 attenuates high-fat diet-induced obesity and insulin resistance in vitro and in vivo. It also regulates hepatic mRNA levels that are associated with glucose and lipid metabolism and insulin response signaling. Further studies to explore the specific mechanistic actions of Ln4 by analyzing changes of microbiota composition should be helpful in better understanding the potential benefits of Ln4, and clinical trials are also needed for developing them into commercial probiotic agent.

## 5. Conclusions

Our findings suggest that *Lactobacillus plantarum* Ln4 that were isolated from Kimchi have the outstanding ability to attenuate diet-induced weight gain and insulin resistance among hundreds of LABs that were isolated from fermented foods. This beneficial effect of Ln4 on obesity and insulin resistance results in recovery of the associated proteins that controlled glucose and lipid metabolism. In addition, Ln4 administration contributes alleviation of fatty liver, hyperlipidemia, and abnormal hepatic mRNA levels that are associated with glucose and lipid metabolism by HFD feeding. Thus, Ln4 has the potential for development as therapeutic probiotics for obesity and type 2 diabetes.

## Figures and Tables

**Table 1:** Specific primer sequence and amplicon size. SREBP1c, sterol regulatory element-binding protein 1c; ACC1, acetyl-CoA carboxylase; FAS, fatty acid synthase; PPARγ, peroxisome proliferator-activated receptor gamma; LDLR, low-density lipoprotein receptor; CD36, cluster of differentiation 36 or platelet glycoprotein 4; AMPK, AMP-activated protein kinase; Akt2, protein kinase Bβ; IRS2, insulin receptor substrate 2; LPL, lipoprotein lipase.

Gene (mouse)	Primer Sequence	Product Size (bp)
SREBP1c	F: 5’- GATGTGCGAACTGGACA-3’ R: 5’-CATAGGGGGCGTCAAACAG-3’	104
ACC1	F: 5’-CCTCCGTCAGCTCAGATACA-3’ R: 5’-TTTACTAGGTGCAAGCCAGACA-3’	103
FAS	F: 5’-AGGGGTCGACCTGGTCCTCA-3’ R: 5’-GCCATGCCCAGAGGGTGGTT-3’	132
PPARγ	F: 5’-TTGATTTCTCCAGCATTTCT-3’ R: 5’-TGTTGTAGAGCTGGGTCTTT-3’	172
LDLR	F: 5’-TGACTCAGACGAACAAGGCTG-3’ R: 5’-ATCTAGGCAATCTCGGTCTCC-3’	118
CD36	F: 5’-CAGATGACGTGGCAAAGAAC-3’ R: 5’-TGGCTCCATTGGGCTGTA-3’	144
AMPK	F: 5’-AAGATCGGACACTACGTCCTG-3’ R: 5’-TGCCACTTTATGGCCTGTCAA-3’	96
Akt2	F: 5’-ACGTGGTGAATACATCAAGACC-3’ R: 5’-ACCCAATGAAAGATCCATCACTC-3’	71
IRS2	F: 5’-TCTACACCCGAGACGAACACT-3’ R: 5’-TGGGCCTTTGCCCGATTATG-3’	103
LPL	F: 5’-TTGCCCTAAGGACCCCTGAA-3’ R: 5’-ACAGAGTCTGCTAATCCAGGAAT-3’	70
β-actin	F: 5’-TGTCCACCTTCCAGCAGATGT-3’ R: 5’-AGCTCAGTAACAGTCCGCCTAGA-3’	101

**Table 2:** Effect of Ln4 administration on HFD-induced dyslipidemia in serum isolated from mice. Five biological replicates were used in each group and represent average ± SEM. The statistical difference was analyzed by one-way ANOVA followed by Tukey’s test. *, **, and *** indicate statistical significance compared with Control (*p* < 0.05, 0.01, and 0.001, respectively). #, and ## means statistical difference between HFD and HFD+Ln4 (*p* < 0.05 and 0.01, respectively). Total TG, total triglyceride; HDL, the high-density lipoprotein; LDL, the low-density lipoprotein.

	Control	HFD	HFD+Ln4
**5-h Fasted Glucose (mg/dL)**	155.2 ± 13.8	230.5 ± 9.3 ***	199.2 ± 3.8 *
**5-h Fasted Insulin (μU/mL)**	18.8 ± 2.8	38.1 ± 1.9 ***	28.4 ± 3.4 ^#^
**Total TG (mg/dL)**	158.2 ± 6.1	203.9 ± 11.1 **	150.8 ± 11.1 ^##^
**HDL-Cholesterol (mg/dL)**	49.2 ± 5.4	79.5 ± 2.0 ***	74.2 ± 2.7 ***
**LDL-Cholesterol (mg/dL)**	21.0 ± 1.9	29.6 ± 1.4 **	30.5 ± 2.1 **
